# *CORR* Insights^®^: Is Model-based Radiostereometric Analysis Suitable for Clinical Trials of a Cementless Tapered Wedge Femoral Stem?

**DOI:** 10.1007/s11999-016-5016-8

**Published:** 2016-08-17

**Authors:** Benjamin Kendrick

**Affiliations:** Nuffield Orthopaedic Centre, Windmill Road, Headington, Oxford, OX3 7LH Oxfordshire UK

## Where Are We Now?

As populations increase in size, and as total hip arthroplasty is increasingly performed in younger patients, the burden on healthcare systems grows. Implant survivorship beyond two decades is becoming an imperative. Unfortunately, many hip implants have been introduced—often as minor modifications of a successful design—only to fail catastrophically. Joint registries pioneered by the Scandinavian countries are powerful tools with which to compare implants in the long-term, but detection of underperforming implants sooner remains difficult because registries generally have focused on the endpoint of revision, rather than on signs of failure that may be evident earlier on. By the time it is apparent that an implant does not have acceptable long-term survival, many thousands, if not tens of thousands, have been implanted.

Radiostereometric Analysis (RSA) has been shown to be adept at detecting implants that are prone to early failure long before the evidence was apparent from longitudinal studies or registry data [2]. However, RSA can be time consuming and labor intensive, and it calls for modified implants. For these reasons, RSA has not become established in many centers around the world. Traditionally, most studies came from those centers (mainly in Europe) where it has long been an integral part of the research program of institutions that have focused on it. This situation has recently changed with the advent of greater computing power and software that accelerates the process, and model-based systems eradicating the need for modified components. But, as with the continuing iteration of hip components potentially leading to a failed design, the modification of RSA must not diminish its accuracy in the quest for it to be easier and quicker.

## Where Do We Need To Go?

A consensus is required on how new implants, or new designs of existing implants, are introduced in to clinical use. In the UK, the National Institute for Health and Care Excellence, in response to the widespread use of poorly performing implants, recommended that surgeons only implant total hip replacements that had solid long-term data, with a minimum survival of 95% at 10 years [3]. This approach, while focusing fully on patient safety, stifles innovation and development. There needs to be a system by which new implants can be evaluated and introduced in a carefully regulated and controlled manner. The call for RSA to evaluate all new implants is not a new idea [4]. But to properly investigate effective evaluation of new implants, there needs to be harmony between the orthopaedic community and the implant manufacturers, with investigations involving research tools such as RSA and good-quality clinical outcome data.

This system should also incorporate laboratory-based testing, and limited clinical release in groups with careful early- and long-term followup to continue in the original cohort. The authors of the current study should be commended for their examination of a new prosthesis. The study is a worthy example of how to: (1) Properly investigate a novel prosthesis, (2) confirm that the results of a clinical RSA trial of the same implant are useful, and (3) provide strong guidance on likely long-term outcome regarding fixation. The strength of this paper is based on its adherence to the consensus guidelines for the use of RSA in orthopaedics [6]. Further papers of this type can only improve our understanding of how implants fail, and help identify those implants that have a long-term future.

## How Do We Get There?

While the current study should be commended, there are still some potential concerns. The system is expensive and arduous for the implant companies and it does not encourage engagement by orthopaedic surgeons unless they are attached to large research centers with the infrastructure to perform the necessary investigation.

The Beyond Compliance system was developed in the UK in response to the NICE guidelines [1]. The system is voluntary on the part of the implant manufacturers, but after the device has been registered and the preclinical testing results have been submitted, the device is released clinically, in a controlled manner. The patients undergo in-depth postmarketing surveillance. This enables any surgeon to register as a Beyond Compliance surgeon and use implants that are newly arrived to the market, obtaining specific consent from each patient for additional followup data to be obtained. The results of this surveillance then directly feeds in to the Orthopaedic Data Evaluation Panel (ODEP), the implant then gains a rating and becomes available for general use [5]. The ODEP rating system has already been adopted in several countries around the world, probably due to its simplicity, but also because of its rigorous nature.

Hopefully, by developing and maintaining a structured, cost-effective, and accessible method of device evaluation, we can avoid repeating the mistakes of the past.
